# Impact of Individual and Worksite Environmental Factors on Water and Sugar-Sweetened Beverage Consumption Among Overweight Employees

**DOI:** 10.5888/pcd11.130207

**Published:** 2014-05-01

**Authors:** Brenda M. Davy, Wen You, Fabio Almeida, Sarah Wall, Samantha Harden, Dana L. Comber, Paul A. Estabrooks

**Affiliations:** Author Affiliations: Wen You, Fabio Almeida, Sarah Wall, Samantha Harden, Paul A. Estabrooks, Virginia Tech, Blacksburg, Virginia; Dana L. Comber, Virginia College of Osteopathic Medicine, Blacksburg, Virginia.

## Abstract

**Introduction:**

The worksite environment may influence employees’ dietary behaviors. Consumption of water and sugar-sweetened beverages (SSBs) affect weight management; however, little research has evaluated the influence of worksite factors on beverage consumption. Our purpose was to determine whether individual and worksite factors are associated with water and SSB intake among overweight and obese employees.

**Methods:**

Data were collected as part of baseline assessments for a worksite-based, weight-management intervention trial. Height and weight of participants (N = 1,482; 74% female; mean age = 47 y [standard deviation (SD) = 11y]; mean weight = 208 lbs [SD = 46 lbs]) were assessed, and participants completed a validated beverage intake questionnaire. Environmental characteristics of worksites (N = 28) were audited. A qualitative comparative analysis (QCA) was used to identify worksite conditions that may support healthier beverage intake patterns.

**Results:**

Most participants were white (75% of sample) with at least some college education or a college degree (approximately 82% of sample). Mean water and SSB intake were 27 fl oz (SD = 18 fl oz) and 17 fl oz (SD = 18 fl oz), respectively; SSB intake (191 kcal [SD = 218 kcal]) exceeded the recommended discretionary energy intake. Statistical models did not identify any significant predictors of water intake. Female sex and increasing level of education and household income were associated with lower SSB intake; baseline body weight and greater number of worksite water coolers and vending machines were associated with higher SSB intake. The QCA identified worksite type (ie, not manual labor) as a condition necessary for healthier beverage consumption; a worksite break policy of 2 or more per day may lead to unhealthy beverage consumption.

Lower SSB consumption was noted among older participants, female participants, and among participants with higher education and income levels.

**Conclusion:**

Workplace factors influence beverage consumption among overweight employees. Limiting vending machine availability and implementing policies that promote weight management may improve employee health.

## Introduction

Obesity is a major public health concern in the United States (1), and effective, long-term weight-management intervention strategies are needed. Attention has focused on identifying dietary factors that may promote successful weight management. Americans of all age groups are consuming more daily total energy, and a significant portion of the increase in total energy intake is derived from energy-dense snacks and energy-containing beverages (2).

Consumption of sugar-sweetened beverages (SSBs), including soft drinks, fruit drinks, and energy drinks, is associated with unhealthy weight status (3). Most (ie, >75%) people in the United States aged 2 years or older consume more than 2 servings of SSBs each day (4). Excessive sugar consumption has been associated with adverse health outcomes such as greater energy intake, higher body weight, and lower intake of essential nutrients, and the American Heart Association has recommended minimizing SSB intake to half of one’s discretionary calorie allowance (5). In contrast, water consumption is associated with healthier dietary patterns and a lower overall energy intake (approximately 200 kcal less per day) (6). Increasing daily water consumption facilitates weight loss and weight-loss maintenance among some populations (7,8); replacing 2 servings (approximately 200 kcal/d) of energy-containing beverages per day with noncaloric beverages (ie, water or diet soda) leads to a 2% to 2.5% weight loss in 6 months (9). Therefore, replacement of SSBs with water may be an effective weight management strategy.

To improve public health, it may be necessary to address interactions between individual and environmental influences on energy balance (10). Environmental approaches to improving health behaviors could include settings such as schools, health care systems, and communities. In addition to reaching large numbers of adults (11), worksite-based interventions may augment social support and facilitate adherence to weight-management programs (12). Financial benefits of worksite health-promotion programs include reductions in sick leave, health plan costs, workers’ compensation, and disability insurance costs (13). The worksite food environment can affect food choice (12,14); however, this research has largely focused on increasing and reducing costs of healthy foods and on reducing portion sizes of foods and beverages (14–16). Pricing strategies appear to be more effective for promoting healthier snack than beverage purchases (14–16), although proportional pricing (ie, keeping price per gram consistent across sizes) may reduce large-sized soft drink purchases among overweight and obese employees (15). It remains uncertain how individual and environmental factors influence beverage consumption behaviors (17), particularly in settings such as worksites (18). 

Our purpose was to determine whether individual and worksite factors are associated with water and SSB consumption among overweight and obese employees. We performed a cross-sectional analysis of data from a worksite-based, weight-management intervention trial. In addition to demographic variables and habitual beverage consumption, assessments included worksite environmental characteristics and a qualitative comparative analysis (QCA) to identify worksite conditions that may support healthier beverage intake patterns.

## Methods

### Individual characteristics

Data were collected as part of baseline assessments conducted at 28 small (<300 employees) to medium-sized (300–599 employees) worksites enrolled in a weight-management intervention trial (reach and representativeness reported elsewhere) (19). Baseline data collection for the larger trial began in February 2008 and was completed in May of 2009. Most worksites (approximately 60%) were recruited from rural and urban locations in southwestern Virginia. Other worksites were located in Richmond, Virginia; Norfolk, Virginia; south-central Virginia; and Denver, Colorado. Participants reported demographic factors (ie, sex, age, race, education and income level, occupation), and educational level was categorized as no high school, some high school/high school diploma, some college, college graduate, or postgraduate education. Total annual household income was categorized as less than $29,000, $30,000 to $49,999, $50,000 to $99,999 and $100,000 or more. Height and weight were measured using a WB-110A scale (Tanita Corporation, Tokyo, Japan) and were assessed without shoes, to calculate body mass index (BMI, weight in kilograms divided by the height in meters squared). Individuals were eligible for participation if they were overweight (BMI 25.0–29.9 kg/m^2^) or obese (BMI ≥30 kg/m^2^) (20).

Habitual beverage intake was assessed in eligible individuals using the Beverage Intake Questionnaire (BEVQ), which is a rapid (<5 minutes), valid, and reliable quantitative self-administered tool, with a reading grade level of 6.9 (21). The BEVQ assesses total beverage consumption (in fluid ounces and in calories) and 19 specific beverage categories including water through reported consumption frequency and volume over the past month. SSB consumption was calculated on the basis of reported consumption of the following beverages: sweetened juice beverage/drink, soft drinks, regular sweetened tea, coffee with cream and/or sugar, mixed alcoholic drinks, meal replacement shakes/protein drinks, and energy drinks.

### Worksite characteristics

Environmental factors were evaluated using an observational method assessment by trained evaluators, the Checklist of Health Promotion Environments at Worksites (CHEW) (22). The CHEW was used to assess the following worksite environmental characteristics: number of soft drink vending machines, number of regular soda slots, number of water coolers, and number of water fountains. Additional variables that were not explicitly tied to water or SSBs but that could reflect worksite norms for healthful living (ie, health promotion signage) were also assessed using the CHEW and included in a QCA.

### Statistical analysis

Water and SSB consumption was evaluated for individual factors (baseline weight, BMI, sex, race, education, and income level) and worksite environmental factors (number of vending machines, number of regular soda slots, number of water coolers, number of water fountains, worksite size). Statistical analyses were performed using Stata 12 (StataCorp LP, College Station, Texas). Analyses included descriptive statistics, *t* tests for group differences according to worksite size, multiple linear regression models accounting for data clustering in worksites, multilevel linear mixed models, and Heckman Sample Selection models. Quantile regression models were used to examine heterogeneous impact across outcome distributions.

Because the beverage and water consumption information was available only for those employees who participated in the weight-loss programs, testing and correcting for potential self-selection bias were done to improve external validity inferences relative to any significant predictors for beverage consumption. The testing and correcting were done using the Heckman Sample Selection methods that allow for correlation between individual decisions about participating in the weight-loss program and the individual’s beverage consumption behaviors. To test for the existence of selection bias, χ^2^ tests on the correlation component of the maximum likelihood function were used. No selection bias was detected for SSB consumption, but bias was detected for water consumption. As a result, our tests associated with water consumption account for this bias. We also explored 2 unique ways to account for the clustering of our data (ie, individual data clustered within worksite). First, we used a multiple linear regression model whose standard errors were adjusted to account for worksite clustering. Second, we used 2-level, hierarchical, mixed linear regression models with cluster-robust standard errors to improve the confidence in our findings. All models include individual-level demographic variables (ie, age, sex, race, education, income, and baseline weight) and worksite level characteristics (ie, the numbers of water coolers, water fountains, vending machines, and regular soda slots and the total number of employees at the worksite). A *P* value of less than .05 was considered significant.

### Qualitative comparative analysis

We also conducted an exploratory QCA (23) to determine whether specific environmental characteristics were associated with worksites with the most healthful beverage intake among employees when compared with those with the least healthful intake. QCA can provide preliminary information on the conditions that are necessary (ie, patterns present in all successful cases but also in some unsuccessful cases) and sufficient (ie, patterns present in only successful cases) to achieve a given outcome (24). To complete the QCA, worksites were rank-ordered from highest to lowest on water and SSB intake, using the mean water and SSB consumption reported by employees. Worksites with the combination of highest water consumption (above the mean for all 28 worksites; >28 fl oz) and lowest SSB caloric intake (below the mean for all 28 worksites; <191 kcal) were identified (n = 4; mean daily water intake = 32 fl oz, mean daily SSB caloric intake = 151 kcal) and compared with the worksites with the combination of lowest water consumption and highest SSB caloric intake (n = 5; mean daily water intake = 25 fl oz, mean SSB caloric intake = 243 kcal). A list of 10 worksite conditions that theoretically could influence employees’ beverage consumption habits, which were not captured in the quantitative modeling analysis, was developed for the QCA (Table 1). Data were collected from the worksite or through observation for size, type of labor, break policy, shift work, signage, and on-site canteen or exercise facilities. Data were collected from employees on average length of a workday and the presence of worksite policies that support weight loss. Conditions present were designated by a “1” and not present designated by a “0.”

**Table 1:** Summary Truth Table: Cross-Case Comparison of Worksite Conditions Theoretically Related to Healthier Beverage Consumption in Worksites Enrolled in a Weight-Management Intervention Trial^a^

Worksite Condition	Site Identification								
	Healthiest Worksites				Least Healthy Worksites				
	15	1	22	3	10	18	23	6	25
Beverage intake: highest water/lowest SSB	1	1	1	1	0	0	0	0	0
1. Small worksite size^b^	0	0	1	1	0	1	1	1	0
2. Type of work: manual labor	0	0	0	0	1	1	1	1	0
3. Break policy: ≥2/d	0	1	1	0	1	1	1	1	1
4. Shift work	0	0	1	1	1	0	1	0	0
5. Workday exceeds 8 hours	0	0	0	0	0	0	0	0	0
6. Health promotion signage present	0	1	1	1	1	1	1	1	1
7. Diet signage present	0	0	0	1	1	1	0	1	1
8. On-site exercise facilities	0	0	1	0	0	0	0	1	1
9. On-site canteen	1	0	0	1	0	0	0	0	1
10. Weight management policy^c^	1	0	1	1	0	0	0	1	0

a Conditions present are designated by a “1,” and conditions not present are designated by a “0.”

b Worksites with fewer than 300 employees were considered small.

c Fifty percent or more of employees report that their employer has policies that support their weight-management efforts.

## Results

Of the initial sample enrolled (N = 1,780), only people with complete data on beverage intake were used in this analysis. Because of missing information on regular soda slots for 2 worksites, our final sample sizes were n = 1,482 for the beverage consumption model and n = 1,476 for the water consumption model. Most participants were female (74%) and white (75%), with some college education or a college degree (82%) (Table 2). Mean age was 47 years (standard deviation [SD] = 11 y [range, 20–86 y]), and the mean weight of participants was 208 lbs [SD = 46 lbs]. A significant portion of the participants (37%) reported an annual household income below $50,000. Mean SSB and water intake were approximately 17 fl oz and 28 fl oz, respectively (Table 2).

**Table 2:** Participant and Worksite Descriptive Characteristics, 28 Small to Medium-Sized Worksites Enrolled in a Weight-Management Intervention Trial

Variable	Full Sample	Small Worksites^a^	Medium-Sized Worksites^b^	*P* Value^c^

	Mean (Standard Deviation)			
Age, y	46.6 (10.9)	46.6 (11.0)	46.6 (10.9)	.94
Female, %	74.2 (43.8)	68.4 (46.5)	81.2 (39.1)	<.001
White, %	75.0 (43.3)	73.2 (44.3)	77.3 (41.9)	.07
High school or less education, %	17.9 (38.4)	12.5 (33.1)	24.6 (43.1)	<.001
Annual household income ≥$50,000, %	63.2 (48.2)	59.9 (49.0)	67.3 (47.0)	.004
Body weight, lbs	207.9 (46.5)	212.8 (48.1)	201.9 (43.7)	<.001
Body mass index, kg/m^2^	33.2 (6.5)	33.6 (6.7)	32.7 (6.3)	<.001
No. of water coolers	3.8 (6.1)	3.0 (6.5)	4.7 (5.6)	<.001
No. of water fountains	7.8 (7.4)	5.1 (4.5)	11.1 (8.8)	<.001
No. of vending machines	6.7 (4.9)	5.6 (2.3)	8.1 (6.7)	<.001
No. of regular soda slots	43.5 (62.0)	33.4 (26.9)	55.9 (85.9)	<.001
No. of employees	307.7 (107.6)	234.9 (42.5)	396.9 (95.4)	<.001
SSB intake, fl oz	16.8 (18.0)	17.8 (18.7)	15.5 (17.1)	.01
Water intake, fl oz	27.5 (17.9)	28.1 (18.2)	26.7 (17.5)	.13

Abbreviation: SSB, sugar-sweetened beverage.

a Small worksites have fewer than 300 employees.

b Medium sized worksites have 300 to 599 employees.

c *P* values calculated using *t* test.

Fifteen worksites were classified as small (<300 employees) and 11 were classified as medium-sized (300–599 employees). Demographic differences, except for age and water intake, between small and medium-sized worksites were noted (Table 2). We observed substantial variation in the worksite beverage environment; the number of water coolers ranged from 0 to 24, the number of water fountains ranged from 0 to 32, the number of vending machines ranged from 0 to 25, and the total number of regular soda slots ranged from 0 to 289.

Results from the regression analysis are presented in Table 3. The detailed results focus on the beverage model and the first stage of the Heckman model (ie, the program participation equation), which are not reported in the table but can be obtained from the primary author (B. M. D.). The likelihood ratio test of selection bias was significant (*P* = .003). When the models were corrected for this bias, none of the individual and worksite characteristics were associated with water consumption.

**Table 3:** Multiple Regression Models: Sugar-Sweetened Beverage (SSB) and Water Intake of Employees Participating in a Worksite-Based Weight-Management Intervention Trial

Characteristic	SSB Model 1: Linear OLS Model		SSB Model 2: Multilevel Model		Water Model 3: Heckman Sample Selection Model	
	β (Robust SE)	*P* Value	β (Robust SE)	*P* Value	β (SE)	*P* Value
Age	−0.13 (0.05)	.01	−0.13 (0.05)	.006	−0.07 (0.06)	.24
Female	−3.66 (1.42)	.01	−3.66 (1.41)	.01	0.85 (1.53)	.58
White	−0.32 (0.88)	.72	−0.32 (0.88)	.72	0.08 (1.65)	.96
Education^a^	−4.01 (1.15)	.002	−4.01 (1.15)	<.001	−0.002 (1.70)	>.99
High income^b^	−2.99 (1.18)	.02	−2.99 (1.17)	.01	2.38 (1.53)	.12
Baseline weight	.03 (0.01)	.01	0.03 (0.01)	.007	0.01 (0.01)	.36
No. of water coolers	0.25 (0.06)	<.001	0.25 (0.05)	<.001	−0.01 (0.10)	.91
No. of water fountains	−0.05 (0.07)	.48	−0.05 (0.07)	.47	−0.13 (0.10)	.19
No. of vending machines	0.43 (0.19)	.03	0.43 (0.22)	.02	−0.12 (0.31)	.69
No. of regular soda slots	−0.01 (0.01)	.32	−0.01 (0.01)	.31	0.02 (0.02)	.30
No. of total employees	−0.01 (0.01)	.21	−0.01 (0.01)	.19	−0.01 (0.01)	.43
No. of observations	1,482		1,482		1,476^c^	
LR test of Model 2 vs Model 1	χ^2^ (1) = 0.00; *P* value = >.99				NA	
LR test of selection bias	χ^2^ (1) = 0.09; *P* value = .768				χ^2^ (1) = 8.98; *P* value = .003	

Abbreviations: SE, standard error; OLS, ordinary least squares; LR, likelihood ratio; NA, not applicable.

a = 1 if education is at or beyond college level.

b = 1 if income is at or beyond $50,000.

c Observations differ from the SSB models because 6 individuals did not report water intake. Total observations of stage 1 of the Heckman selection model = 4,666.

The likelihood ratio test of self-selection indicated no self-selection bias in the beverage consumption equation (*P* = .77); therefore, we used standard multiple linear regression models (Table 3). SSB Model 1 is the linear ordinary least squares regression model with cluster-robust standard errors. The SSB Model 2 is the multilevel hierarchical model with cluster-robust standard errors. Both models showed consistent findings related to predictors of SSB consumption.

Lower SSB consumption was noted among older participants, female participants, and participants with higher education and income levels. Participants with postcollege education consumed approximately 4 fl oz less SSBs per day than those with less than a college education, and those with household income at or above $50,000 consumed approximately 3 fl oz less SSB than those with income of $49,999 or less. Furthermore, higher baseline weight was associated with a higher beverage intake.

In the worksite beverage environment, given the same number of employees (ie, holding the worksite size constant), greater water cooler and vending machine availability were associated with higher SSB consumption. A follow-up analysis was conducted to determine whether the percentage of total beverage vending machine slots devoted to regular sodas (% of regular soda slots) was associated with SSB intake, instead of the total number of regular soda slots. The percentage of regular soda slots variable was not a significant independent predictor of SSB intake in the linear or multilevel models (β = 5.47, *P* = .11 and .10, respectively). Overall findings were unchanged, except that vending machine number was no longer a significant predictor of SSB intake with inclusion of the percentage of regular soda slots variable in the models.

The only condition identified as necessary for “healthier beverage” consumption (Table 1) was that the worksite type is not manual labor (ie, packing, shipping, maintenance, and assembly); having a break policy of 2 or more per day may lead to unhealthier beverage consumption. Perceived worksite weight management policies were present in 3 of the 3 “healthier beverage” worksites and in only 1 of the 5 least healthy beverage worksites. No patterns for sufficient conditions were identified in the QCA.

## Discussion

We did not identify any individual or worksite factors in our sample of overweight and obese employees that independently predicted water consumption. However, both individual and worksite factors were independently associated with SSB consumption. A lower intake of SSB was noted among older participants, those with higher levels of education and income, and women, whereas baseline body weight and a greater number of worksite water coolers and vending machines were positively associated with SSB intake. The finding related to water coolers and SSB intake was surprising. Certain worksites may have been more likely to have water coolers as a result of a warmer work environment, which is more likely to be present in manual labor sites; manual labor worksites are more likely to have employees who have lower educational and income levels and are, therefore, more likely to consume SSBs. This unexpected finding may be consistent with our QCA results, which identified worksite type (ie, not manual labor) as a condition necessary for healthier beverage consumption, and those of Levy et al (25) who reported that employees in job categories requiring less education (eg, service workers) were more likely to purchase unhealthy foods such as sugary beverages. Similar to findings reported by Escoto et al (26), findings from our QCA did not identify longer workdays or workweeks (ie, > 40 hours/week) as being associated with unhealthier beverage consumption among employees. However, frequent work breaks may promote unhealthy beverage consumption patterns (ie, less water and more SSBs), and having a worksite policy that employees reported as supporting their weight-management efforts may lead to healthier beverage consumption patterns. Thus, workplace factors do appear to influence beverage consumption among overweight employees.

Diet quality is generally associated with socioeconomic standing (27). Adults with less education are less likely to consume healthful diets and more likely to be overweight or obese, and those with low income and education are more likely to consume diets high in sugars (28). However, obesity prevention and treatment research has extended beyond the individual level to include environmental factors that influence weight-related behaviors (15). For example, the rise in food availability and accessibility coupled with an increase in sedentariness appears to be a prime driver of the obesity epidemic (10,17). Our findings could be of interest to those developing worksite-based interventions targeting improvements in beverage consumption patterns and reductions in body weight and possibly health plan costs. Additional strategies for improving beverage intake behaviors consistent with the US dietary guidelines (replace sugary drinks with water) and American Heart Association guidelines (limit added sugar intake) could include removing price benefits for larger sizes (ie, proportional pricing) in worksite vending machines and cafeterias and increasing availability and reducing prices of healthier alternatives to SSBs. Vermeer et al (15) reported that overweight or obese consumers were less likely to choose large soft drink sizes when proportional pricing strategies were used; 67% of overweight or obese individuals chose a medium-sized soft drink and 0% chose a large-sized soft drink with proportional pricing, compared with 33% who selected large-sized soft drinks when value size pricing was used. Reducing the cost of healthy beverages (defined by these authors as beverages with less than 50 kcal) and snack items by 10% or more and increasing availability of healthy items by 50% in worksite vending machines led to increases in sales of healthy items, although results appeared more effective for snack than beverage purchases (14). Our findings suggest that reducing access to vending machines could encourage healthier drink choices. These findings also suggest that the health effects of worksite environmental and policy changes warrant further evaluation — specifically, the role of water cooler and vending machine availability and the frequency of work breaks.

Strengths of this investigation include a large sample size, detailed information on habitual beverage consumption using a validated tool, and evaluation of both individual factors and the worksite environment. We acknowledge several limitations. First, the population consisted of overweight and obese adults; therefore, beverage patterns were not compared with those of people with normal weight status and may not apply to the general population. Furthermore, the population predominantly consisted of white women, which also limits the generalizability of our results. Studies of nationally representative samples have identified demographic factors associated with water consumption, specifically, age and income level (29) and weight status (30). Thus, the lack of association of individual factors with water consumption could be attributed to our population being homogenous with respect to weight status (ie, primarily those who were overweight or obese), age, and income level. Information on caffeine’s role in beverage selection was not evaluated, and habitual beverage consumption was self-reported. Finally, the cross-sectional nature of this investigation precludes us from drawing conclusions about causality.

This investigation represents the first extensive evaluation of the workplace environment and its influence on water and SSB consumption. Multicomponent interventions that target changes aimed at individual and environmental levels may be necessary to control overweight and obesity among adults in worksite settings, as well as in other settings such as schools or communities. As an example, Muckelbauer et al (31) conducted an intervention focused on promoting and providing drinking water to elementary school children. The intervention included educational messages delivered in the classroom by teachers and increasing water fountain availability in schools and was effective in decreasing the risk of overweight and in increasing water consumption during a school year. Furthermore, this intervention focused on schoolchildren in “socially deprived” urban areas of Germany. Process evaluation and a 19-month follow-up assessment determined the intervention to be sustainable and feasible (32). Although policy changes will not entirely solve the obesity epidemic, multicomponent interventions to improve beverage consumption behaviors and health outcomes that target individuals and their environment appear warranted. Future intervention trials are warranted to determine whether reducing SSB consumption and increasing water consumption could be an effective dietary strategy for worksite-based weight management interventions and whether individual and environmental intervention features mediate or moderate intervention effectiveness.
